# Development and Limitations of Exposure Biomarkers to Dietary Contaminants Mycotoxins

**DOI:** 10.3390/toxins13050314

**Published:** 2021-04-28

**Authors:** Paul C. Turner, Jessica A. Snyder

**Affiliations:** Maryland Institute for Applied Environmental Health, School of Public Health, University of Maryland, College Park, MD 20742-2611, USA; jasnyder@umd.edu

**Keywords:** aflatoxin, biomarkers, deoxynivalenol, fumonisin, ochratoxin A, urine, blood

## Abstract

Mycotoxins are toxic secondary fungal metabolites that frequently contaminate cereal crops globally, presenting exposure hazards to humans and livestock in many settings. The heterogeneous distribution of mycotoxins in food restricts the usefulness of food sampling and intake estimates for epidemiological studies, making validated exposure biomarkers better tools for informing epidemiological investigations. While biomarkers of exposure have served important roles for understanding the public health impact of mycotoxins such as aflatoxins (AF), the science of biomarkers must continue advancing to allow for better understanding of mycotoxins’ roles in the etiology of disease and the effectiveness of mitigation strategies. This review will discuss mycotoxin biomarker development approaches over several decades for four toxins of significant public health concerns, AFs, fumonisins (FB), deoxynivalenol (DON), and ochratoxin A (OTA). This review will also highlight some knowledge gaps, key needs and potential pitfalls in mycotoxin biomarker interpretation.

## 1. Introduction to Mycotoxins

The evolution of humans from hunter gatherer to cultivator, approximately 10,000 years ago, may have opened a window of opportunity for many families of secondary fungal metabolites to be frequent companions in our diets [1]. Many of these compounds are proven to be highly toxic in veterinary settings and animal models [1,2,3], launching the naming of these secondary metabolites as mycotoxins. Among the hundreds of mycotoxins identified, several are contaminants in key dietary staples at levels of contamination, frequency of contamination, and regularity of consumption to be of public health concern [1,2,3,4,5]. Estimates of the frequency of agricultural crops that are contaminated range from approximately 25% to 80% [2,5] with grains, nuts and some fruit crops being particularly susceptible to certain families of mycotoxins. Mycotoxins of major public health concern include aflatoxins (AFs) produced from *Aspergillus* species, fumonisins (FBs), deoxynivalenol (DON) from *Fusarium* species, and ochratoxin A (OTA) produced by both *Aspergillus* and *Penicillium* species [2]. AFs and FBs are typically more frequent contaminants of crops in hot and humid climates such as Central America, tropical Asia and sub-Saharan Africa, where staple foods such as maize and groundnuts (peanuts) are often contaminated. For AFs, both field growth and long-term storage contribute to the burden of contamination, while FBs are predominantly a field-produced toxin of maize [2]. DON is more prevalent in temperate climates on grains such as wheat and maize, and also tends to accumulate in the field to a greater extent than during dry storage, while OTA is found on a variety of grains, fruits and coffee and can be produced both in the field and during storage. The production of mycotoxins is favored within a distinct set of temperature ranges and relative humidity, though mixtures of mycotoxins are increasingly being reported. Mycotoxins tend to be resistant to processing, and their stability during cooking also contributes to dietary exposure [2,3]. More developed regions of the world tend to have both regulation and the infrastructure to enforce such regulation, while the infrastructure in developing-world regions is lacking to support regulation, especially in subsistence farm settings. Thus, individuals that are particularly vulnerable face a combination of limited dietary variation and heavy reliance on one or two high-risk dietary staples.

With the exception of the AFs, mycotoxins as a group of contaminants remain a mostly poorly examined global health issue, despite the predicted high frequency of exposure and the demonstrated animal toxicities [1,4,5,6]. AFs are potent liver toxins, human carcinogens [4,7,8,9], and suspected human growth modulators; and in animals, cause cancer and affect growth and immune function [4,10,11]. FBs are suspected human carcinogens and have been recently found to be postulated growth modulators; and in animals, cause diverse toxicity including cancer, neural tube defects, equine leukoencephalomalacia and porcine pulmonary edema [1,4]. DON has effects on the GI tract and immune system of animals, and is suspected to cause growth faltering [1,2,3]. OTA is a suspected non-neoplastic and developmental nephrotoxin in humans [12].

The heterogeneous distribution of mycotoxins in the diet has restricted more classical epidemiological approaches, partly because these studies struggle to provide good estimates of exposure. However, the development, validation and use of exposure biomarkers offer improved exposure assessment. This short review is aimed at groups engaged in capturing and interpreting biomarker data. However, it is neither an exhaustive review of every mycotoxin biomarker survey nor does it review epidemiological data generated by biomarkers. Rather, it highlights several major developments in an attempt to better estimate exposure to AFs, FBs, DON and OTA; and it examines some limitations, and outlines some data gaps.

## 2. Exposure Biomarkers

There have been significant strides in approaches to better capture exposure data in settings where more traditional epidemiological approaches are limited. For the mycotoxins, two basic food contamination processes can be envisaged. Firstly, contamination may come from one or two very limited dietary or fungal sources, e.g., for AF and FB, but the distribution in these foods is highly heterogeneous, creating difficulty in capturing exposure by food measures alone, food diaries or questionnaires alone, or a combination of these approaches. Dietary contamination of significant public health concern is also more likely in lower-resource settings and thus sampling from either food stores or plate-ready food is potentially culturally complex, with reasonably representative sampling likely to be overly burdensome to family food security. The second scenario is that multiple food sources are contaminated, as is seen more frequently for DON and OTA, but again with a heterogeneous distribution of contamination in the various foods. In this scenario, food diaries or questionnaires provide good information that exposure occurred, but lack quantitative estimates at the individual level. Measuring body fluids or tissues allows information to be captured accurately on the concentration of either the parent toxin or a metabolite from multiple sources, and thus potentially provides a better estimate of exposure. For the mycotoxins discussed, we will highlight studies where a biological measure has identified the toxin, or a metabolite, indicative of “some level” of exposure, with particular attention given to studies where attempts to understand dose–response relationships between the putative biomarker and the estimated intake are additionally reported.

## 3. Aflatoxins

**Figure 1:**
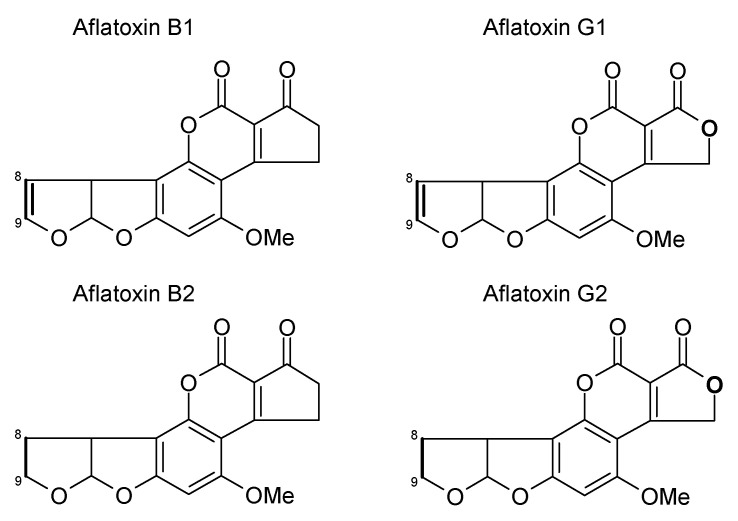
Structures of the four naturally occurring aflatoxins. The 8,9 position is where the reactive epoxide can be readily formed across the double bond.

**Figure 2:**
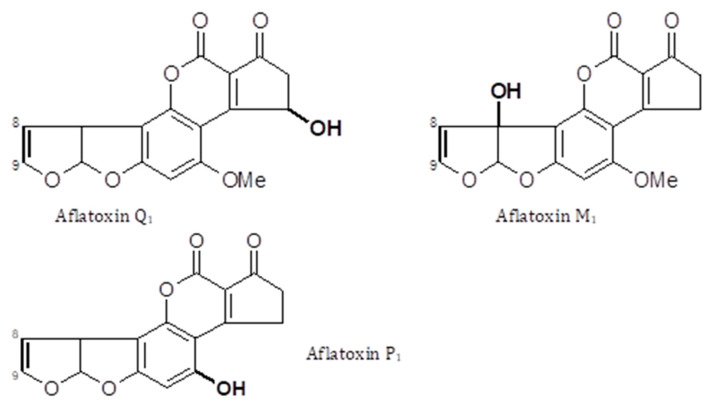
Structures of aflatoxin metabolites aflatoxin Q1, aflatoxin M1, and aflatoxin P1, highlighting phase 1 hydroxylation reactions.

**Figure 3:**
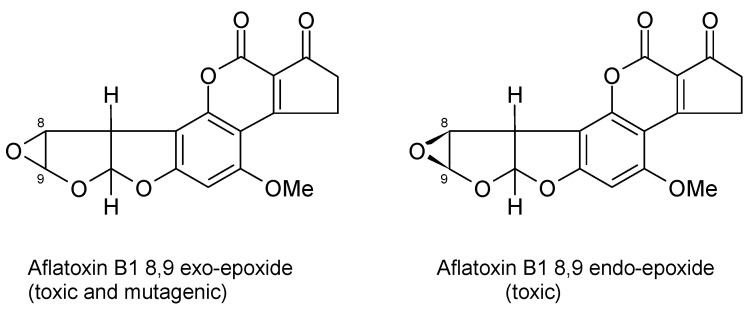
Structures of the aflatoxin B1 exo- and endo-epoxides.

**Figure 4:**
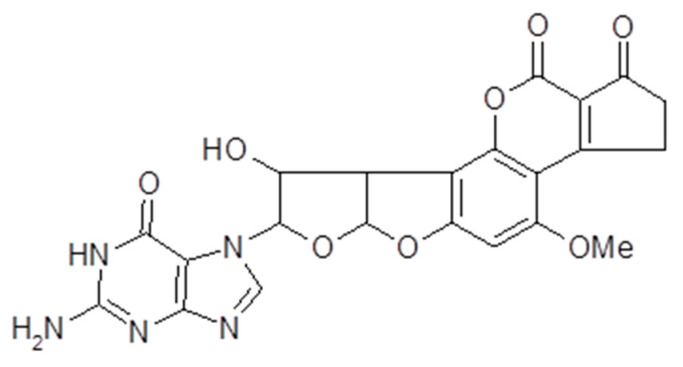
Structure of aflatoxin-N7-guanine. Formed by the aflatoxin exo-epoxide binding to guanine in DNA.

AFs are a family of closely related toxins that include AFB1, AFB2, AFG1 and AFG2 (Figure 1). All four of these toxins have been observed in the urine, indicative of exposure. However, a useful quantitative relationship between the intake and the urinary concentration that would support exposure assessment has not been demonstrated. Instead, an understanding of the toxicokinetics of AFs has helped the development of AF exposure biomarkers [13,14,15]. Among the naturally occurring aflatoxins, AFB1 occurs most frequently, is the most toxic and carcinogenic, and remains the most studied mycotoxin to date. AFB1 is metabolized predominantly in the liver by a number of cytochrome P450 enzymes [13,14], generating several hydroxy-metabolites, including AFM1, AFQ1 and AFP1 (Figure 2), and two highly reactive epoxides, AFB1 exo-8,9-epoxide and AFB1 endo-8,9-epoxide (Figure 3). The epoxides are short lived but highly reactive, and form covalent adducts with multiple macromolecules including DNA and proteins [15,16,17,18,19,20]. Aflatoxin B1 exo-epoxide preferentially forms an adduct with DNA at the N-7 position of guanine residues, which can subsequently undergo depurination, releasing AF-N7-guanine (AF-N7-Gua) (Figure 4) [13,15].

A series of studies have examined the relationship between AF ingestion and urinary measures indicative of exposure [16,17,18,19]. In one study, 20 Gambian adults were followed for a period of seven days, during which their daily ingestion of AF was captured by daily plate-ready food sampling, while on days 4–7, urinary samples were analyzed for AF and AF metabolites [16]. Daily food measures revealed a typical two to three log variation in ingestion per day on any given day examined. Authors reported an integrated measure of typical daily intake against total urinary AF. This was measured using a non-specific AF enzyme-linked immunosorbent assay (ELISA), i.e., one that could capture multiple AF metabolites. This approach revealed a highly significant correlation between AF intake and total urinary AF (*p* < 0.001, r = 0.65). A more detailed urinary analysis revealed multiple AF species including AFG1, AFP1, AFQ1 and AF-N7-Gua, and in refined regression analysis, AF ingestion was most strongly associated with AF-N7-Gua (*p* < 0.0001, r = 0.82).

In a similarly designed study involving individuals from Guangxi Autonomous Region, People’s Republic of China [17], a 7-day food collection and 6 day urinary collection were also used. Here, the integrated AFB1 intake versus urinary AF-N7-Gua comparison revealed a highly significant association (*p* < 0.0001, r = 0.80, *n* = 30), while the previous-day intake versus the individual urinary measure gave a similar albeit slightly less significant association (*p* < 0.0001, r = 0.65, *n* = 120). In the latter regression analysis, authors used data for a combined four-day urinary dataset rather than for the individual daily urine for each of the four days collected, thus partially smoothing for daily fluctuation. Samples from the above study had previously been used to examine the relationship between AFB intake using thin layer chromatography (TLC), and urinary AFM1 (using an ELISA) looking at the previous-day and current-day intake against the urinary measures and, again, a highly significant correlation (*p* < 0.0001, r = 0.66) was reported [18]. Groopman and colleagues re-examined these data [19] using high performance liquid chromatography (HPLC) and reported a similar outcome, albeit looking at the previous day only and the urinary measure (*p* < 0.0001, r = 0.55). These studies combined indicated that understanding and describing specific metabolites were key to the development of exposure assessment tools for AFs. They indicate that both AF-N7-Gua and AFM1 in urine are strongly correlated with intake and that both are regarded as good biomarkers of relatively recent AF exposure. AF-N7-Gua has additional utility as it would be predictive of being on the causal pathway of AF-induced hepatocellular carcinoma [8,9,19,21], the primary disease for which the AF biomarkers were being developed to establish causality. Neither urinary AFB1 nor other AFB1 metabolites such as AFP1 or AFQ1 have been demonstrated to be positively correlated with the intake of AFB1 [16,17,19]. In fact, an inverse relationship between the urinary measure of AFB1 and AF intake was demonstrated which would confound any attempt to use this measure in epidemiology or within mitigation studies. For this reason, urinary AFB1 is informative to some extent that exposure occurred, but does not provide a useful quantitative indicator of exposure.

**Figure 5:**
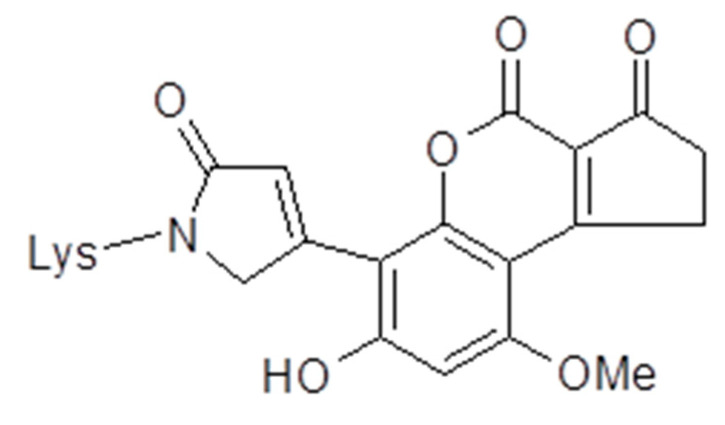
Structure of aflatoxin B-lysine (AF-lys). The major Pronase digest product from the aflatoxin–albumin adduct.

While only the exo-epoxide forms guanine adducts in DNA and is regarded as mutagenic [7,8,9], both the endo- and exo-epoxide of AFB1 are toxic and lead to the formation of aflatoxin–albumin (AF-alb) in hepatocytes, and are observable in the sera of exposed animals and humans [20,22,23,24,25,26,27,28,29,30]. As such, albumin adducts of aflatoxins were anticipated as highly informative of hepatoxicity, with perhaps similar value as a biomarker of AF-N7-Gua in urine despite not being on a causal pathway to disease. Typically, the concentration of AF-alb is measured following enzymatic digestion of the protein, and quantitation of the dominant AF-bound amino acid in albumin, aflatoxin-lysine (AF-lys) (Figure 5). Thus, pg AF-lys/mg of albumin, and AF-alb (pg/mg) are often used in the literature as equivalent measures of exposure. However, in this paper, the terminology AF-alb (pg/mg) will be used exclusively. In one study of an association between AF intake and the serum AF-alb, individuals from Guangxi Autonomous Region, People’s Republic of China, used 7 day food collection with six days of concurrent urine collection and blood collection on the seventh day to examine the association (n = 42) [23]. The integrated AFB1 intake versus the single serum AF-alb measured by radioimmunoassay (RIA) revealed a highly significant association (*p* < 0.0001, r = 0.69). It was also noted that average urinary AFM1 over the study period was strongly correlated with the single serum AF-alb measure (*p* < 0.0001, r = 0.60). In a similar study in The Gambia [24], involving 7-day collection of AFB intake from plate-ready food and a measure of serum AF-alb on day 8, a correlation between average AF intake and serum AF-alb (measured by ELISA) was revealed (*p* < 0.05, r = 0.55). In the study by Wild et al. [24], 34 of the samples were additionally measured by an HPLC fluorescence assay (novel at that time), with excellent comparison of the biomarker (*p* < 0.001, r = 0.97), data supportive of the initial observation by ELISA. In contrast to the urinary measures discussed above, and based on the half-life of albumin, the AF-alb biomarker has particular utility as a biomarker as it represents an integrated assessment of exposure over a period of two to three months [28], and thus to some extent smooths for potential daily timing and fluctuation in AF intake. A further advantage of the long half-life of the adduct is that in chronically exposed individuals, the concentration is approximately 30-fold higher than would be obtained from a single exposure, greatly facilitating detection capacity; capacity needed as fmol/pmol quantities measured in assays are significant [28].

The studies to date have been based on settings with frequent chronic exposure in adults. Only one group of researchers has attempted to further examine the relationship between the AF-alb biomarker and AF intake in children [30]. The study in Tanzania involved a recruitment phase and then included two collection periods, approximately 9 months apart, with young children aged 12–22 months at the first blood collection time point. Daily AF intake was estimated over a two-day survey at each of the blood collection time points. Overall, a modest but statistically significant correlation was observed with the AF-alb biomarker and maize-based AF intake for all data (*p* < 0.01, r = 0.43, *n* = 296). It was noted that a limitation in this analysis was that maize was regarded as the primary AF source, while groundnut consumption was not included in the AF estimate. When separately comparing the regression analyses at the two time points, there was an approximate 50% lower groundnut consumption frequency at the first, and a somewhat stronger correlation between maize-based AF intake versus the AF-alb biomarker was reported (*p* < 0.01, r = 0.51, *n* = 148) at that time point. Preliminary observation reveals that groundnuts in Tanzania can be a very significant source of dietary AF exposure in infants, even when consumed in significantly lower amounts than maize [31]; thus, we might predict, but it remains unproven, a stronger relationship if total AF had been measured in the young children validation study [30]. An extension of this study in children was conducted involving 84 infants with matching AF-alb, maize-based AF intake and additionally urinary AFM1 measurements [32]. These data revealed a significant correlation between both urinary AFM1 and estimated AF intake (*p* < 0.001, r = 0.47, *n* = 168) and between urinary AFM1 and AF-alb (*p* < 0.001, r = 0.49, *n* = 168). The comparison with intake again had the caveat that the groundnut contribution to AF intake was not captured. Overall, in high-risk regions of the world, greater than 95% of individuals tested are positive for AF-alb, with concentrations typically spanning a 3-log range, from approximately 3–5pg/mg albumin to >1000 pg/mg, while more developed regions rarely have detectable levels of the biomarker [6,8,9,10,21,25,26,28,33].

## 4. Fumonisin

**Figure 6:**
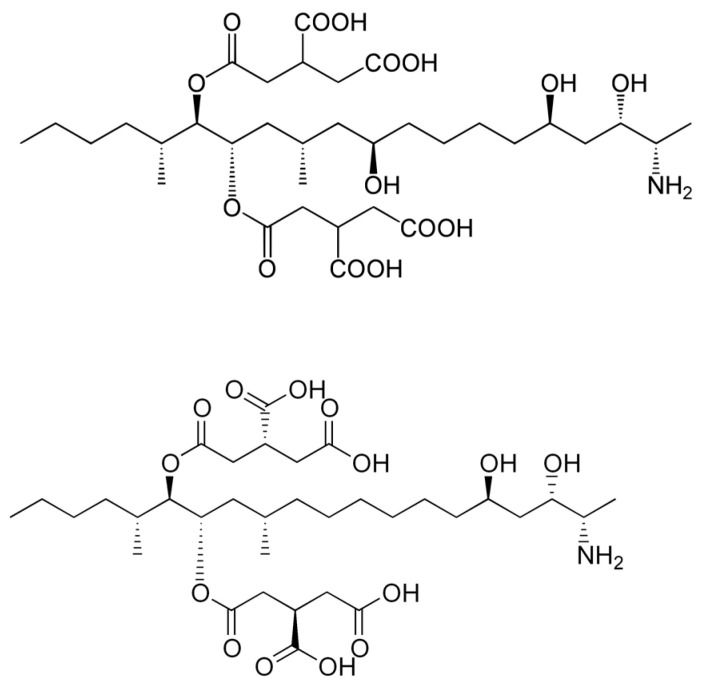
Structures of fumonisin B1 (upper) and fumonisin B2 (lower).

The development of highly affective exposure biomarkers for fumonisin has proven more complex than for aflatoxin. The fumonisins are a family of toxins, with FB1 and FB2 (Figure 6) the most frequently observed, and typically with FB1 representing approximately 70% of the natural contamination of maize [1,2,3,4]. To date, most studies have focused on FB1. Possessing both multiple carboxylic residues and a primary amine means that FB1 in aqueous medium of varying pH is typically charged, thus there is limited uptake of ingested FB1 compared to other mycotoxins discussed in this review [34,35]. FB1 has a sphingoid backbone, and the potential for ceramide synthase inhibition by FB1 was recognized and then rapidly demonstrated in experimental animal models [36,37,38,39,40,41]. Inhibition was demonstrated as a modulation of two physiologically important precursors in sphingolipid production, sphinganine (Sa) and sphingosine (So), leading to an increase in the ratio of Sa/So [36,37,38,39,40,41]. This mechanism appears to be on the causal pathway for toxicity [38,39,41]; somewhat analogous to AF-N7-Gua as a marker of exposure in AF and mutation events leading to liver cancer [4,8,9]. This alteration in Sa/So appears specific for FB1, with perhaps the exception of *alternaria* toxins [40,41]. Thus, a biomarker involving this mechanism was highly attractive for epidemiological purposes, and several groups initiated trials to examine dose–response relationships between FB intake and the suggested elevation in Sa/So ratio.

In one study, following the identification of a high-risk village in Burkina Faso, a survey of 20 husband–wife pairs aged 20–40 was conducted [42,43]. Plate-ready food was sampled over three consecutive days, and the Sa/So ratio was determined in urine and buccal cells on days two to four, and serum on days one and four. No statistically significant relationship was found between FB intake and either urinary or buccal cell Sa/So, while only a modest correlation albeit non-significant was reported between FB intake and serum Sa/So (*p* = 0.06, R^2^ = 0.21). It was noted that the mean intake was <1.0 ug/kg bw day (max 5.2 ug/kg bw/day) during this validation attempt compared with the pilot research, in which an estimated mean FB intake of 10 ug/kg bw/day (max 26 ug/kg bw/day) was identified [42]. This reduced range of exposure during the validation phase limited the power of the study to examine any putative relationship and highlights the difficulty of such studies where variation in contamination is common from year to year. A second study investigated the relationship between FB intake and urinary Sa/So [44], and found significantly (*p* < 0.001) higher Sa/So in high maize consumers (North Argentina and South Brazil combined *n* = 123, Sa/So = 1.27 ± 0.33) compared to a no/low maize consuming regions (South Italy and Central Argentina combined *n* = 42, Sa/So = 0.36 ± 0.02). However, study design issues surrounding the estimates of FB contamination of the maize in the high-risk group, and thus FB intake, and the inclusion of both children and adults in this study, hinder further useful data interpretation. A third study in South Africa [45] examined Sa/So in singleton spot urine (*n* = 55) and in blood (*n* = 41) against estimated FB intake by measuring household stored maize. There were no strong associations between FB intake and either putative biomarker (r = 0.06, *p* > 0.05 and r = 0.11, *p* > 0.05, respectively). When data were dichotomized into high- and low-exposure groups with a greater than 10-fold difference in intake, there remained no significant differences in the urinary and plasma Sa/So ratio, *p* > 0.05 for both. These data were in agreement with a less sophisticated pilot survey in this region [46], that lacked the individual intake data for the comparison. Thus, in contrast to the strong associations observed in animals [36,37,40], modulation of Sa/So by FB was proving either elusive or non-informative in humans. Most animal models used doses that were higher than those reflective of typical human FB exposures. Thus, ceramide synthase may be the molecular target from human exposures, but our typical “high” FB1 exposure may simply not meet a threshold where Sa/So modulations are sustained sufficiently to be readily detectable by this approach. The temporal nature of lower levels of FB1 ingestion versus the modification in Sa and So is also poorly understood. Thus, in this setting, these short-term measures of FB intake may not capture a sufficient time frame.

Overall, these human studies have treated intake simply as “FB” rather than distinguishing between the two main dietary FBs, FB1 and FB2. These toxins have a similar quantitative effect on ceramide synthase and subsequent Sa/So modification ex vivo [34]. However, in vivo experiments in vervet monkeys indicate that FB1 and FB2 are not equipotent modifiers of the serum Sa/So ratio [47,48], at least in part due to their different toxicokinetics. Using a single dose, FB2 generated a far more prolonged modification of the serum Sa/So, with a larger and later peak concentration, and took three times as long (six weeks rather than two weeks) compared to a single FB1 dose for the Sa/So to drop back below 2.0. Doses used in vervet monkeys were high and it remains unclear whether those observations are relevant to humans. However, it is clear that at-risk populations are exposed to a mix of FB1 and FB2 [4,34,35], and to this point, modeling of the human data with respect to biomarker evaluation regard FB1 and FB2 simply as total FB.

A limited study in terms of numbers or participants suggested that a change in the ratio of urinary Sa/So could be observed when comparing pre-FB exposure Sa/So and post-FB exposure Sa/So, while controlling maize consumption over a one-month period [49]. There was a wide range of estimated FB daily intake (0.5–740 ug/kg bw/day) in the consumption month. However, there was no statistical difference in serum Sa/So between pre- and post-maize consumption. In males only, the ratio of the Sa/So ratios (post/pre) was three-fold higher compared to females and for those in the high- versus low-FB-consumption groups. However, post-exposure, there was not an apparent dose–response effect in the later Sa/So ratio even where differences in intake could be 10-fold; rather, the change in ratio was a consequence of the apparent variation from baseline Sa/So. If such a biomarker approach was adopted, the need for understanding a change in ratio that requires baseline ratio data would severely limit any practical use in epidemiological research.

Toxicokinetic evaluation of FB in various experimental animals demonstrated that the uptake and subsequent transfer of FB to urine was relatively low, at only approximately 0.4–2.0% [50,51,52,53,54,55], with most FB1 ingested directly excreted in the feces. The percentage transferred to urine reflects several days of collection, and often at doses higher than would be observed in humans. An initial investigation aimed to demonstrate the presence of FB1 in human urine. It utilized nutritional data and urine samples collected from a cohort of nearly 1000 women in Morelos County, Mexico. Three groups (*n* = 25 per group) were selected based on maize-produced tortilla consumption. Urinary FB1 was detected by liquid chromatography-mass spectrometry (LC–MS) (limit of detection 20 pg/mL) in 75% of the samples (geometric mean 70.1 pg/mL urine; range: non-detectable to 9312 pg/mL). When comparing individuals in the highest-, medium- and low-consumption groups, a trend was observed for urinary FB1 (positivity was 96%, 80% and 45%, respectively; and geometric mean and 95% CIs were 147 (88, 248), 63 (37, 108) and 35 (19, 65) pg/mL, respectively, *p* < 0.001 [56]. These pilot data prompted a better understanding of the quantitative relationship between FB intake and urinary FB concentration. A survey of two counties in China was undertaken [57]. In Huaian county and Fusu county, mean urinary concentrations of 13,630 pg FB1/mg creatinine (range nd–256,000 pg/mg; median 3910 pg/mg) and 720 pg/mg (range nd–3720 pg/mg; median 390 pg/mg) were reported, respectively. However, no significant correlation between urinary FB1 and estimated intake were found. Their data suggested that FB intakes were at least 3-fold higher in Huaian County, and that approximately 1–2% of the ingested FB was transferred to urine. A study in South Africa attempted to assess the relationship between FB1 ingestion and urinary FB1 concentration using average intake measures from plate-ready food (a maize porridge) over two days and subsequent urine collections on days 2 and 3, respectively. The study additionally attempted to incorporate an intervention phase and again compare estimates of intake with the urinary measure. Overall, a moderate correlation (r^2^ = 0.25, *p* < 0.001) was observed between estimated FB1 intake/kg bw/day and urinary FB1 adjusted for creatinine in a survey in which FB1 exposure had a greater than 2 log variation. In this study, the mean transfer of ingested FB1 to urine was estimated to be 0.075% [58]. The intervention phase appeared to involve pooling, cleaning and re-pooling of the cleaned maize, reducing the FB contamination overall. However, the study design suggests that all plate-ready maize samples would be equivalently contaminated, thus restricting that phase of the study from having a wide range of exposure to support the demonstration of a strong dose–response relationship.

In a highly controlled small-scale study, US-based participants deliberately consumed maize tortillas and biscuits on a regular basis to try to better define the toxicokinetics of FB1 in humans. Initially, eight individuals consumed food items at fixed time points over three days with subsequent follow up of three days in which no FB1 intake occurred. Urine samples were collected each day, including day 0 (prior to the start of the feeding study). The mean overall estimated intake of FB1 was 2.9 (SD 0.5) ug/kg bw/day [59]. Urinary FB1 was not observed on day 0 (no exposure), but was observed on days one through five of the study. The ingested FB half-life was suggested to being close to but less than 48 h, and it was estimated that the range of dose excreted in the urine was 0.1 to 0.9% (*n* = 8), thus a 9-fold variation in percent transferred to urine in a controlled modest dose setting. It was also noted that for each day, the mean FB1 urinary concentration had a SD of approximately the size of the mean on any given day. In one additional individual, a 6 day feeding trial occurred, and here the overall recovery in urine was similar to the 3 day trial, <1.0% transfer, though authors noted the apparent half-life where FB was ingested was 48–72 h [59], slightly longer than that estimated for the shorter trial. Authors conclude that the high variability of the data will make epidemiological research involving FB1 more complex.

In probably the largest study ever to examine the relationship between the intake of a mycotoxin and the urinary measure, Torres et al. [60] report estimated FB intake and urinary measures in 1129 paired samples from three departments within Guatemala. There was more than a 3-log variation in FB exposure, and a highly significant albeit modest correlation between FB intake and urinary FB (*p* < 0.0001, r = 0.26). Riley and colleagues had previously suggested that sphingoid bases were likely rapidly phosphorylated and accumulated in red blood cells [61], potentially creating difficulty exploring the Sa/So ratio as an exposure biomarker, despite FB’s likely involvement within this pathway. In a follow-up to the urinary FB study in Guatemala, rather than measuring serum Sa/So, the ratio of the phosphate adducts (Sa-Pi/So-Pi) was measured and compared to urinary FB1 measures [62]. A statistically significant correlation was observed (*p* < 0.0001, r = 0.49), providing for the first time good evidence in humans that the enzyme ceramide synthase is indeed inhibited in humans naturally exposed to FB1 in their diet. Riley and colleagues concluded that population-based estimates for FB exposure could be made in high-risk settings, but individual data were harder to define. Additionally, Riley concluded that the toxicokinetics of FB1 and FB2 in humans are not aligned, and despite frequent FB2 contamination of maize in their study, FB2 was never recovered from urine in their analysis [60].

## 5. Deoxynivalenol

**Figure 7:**
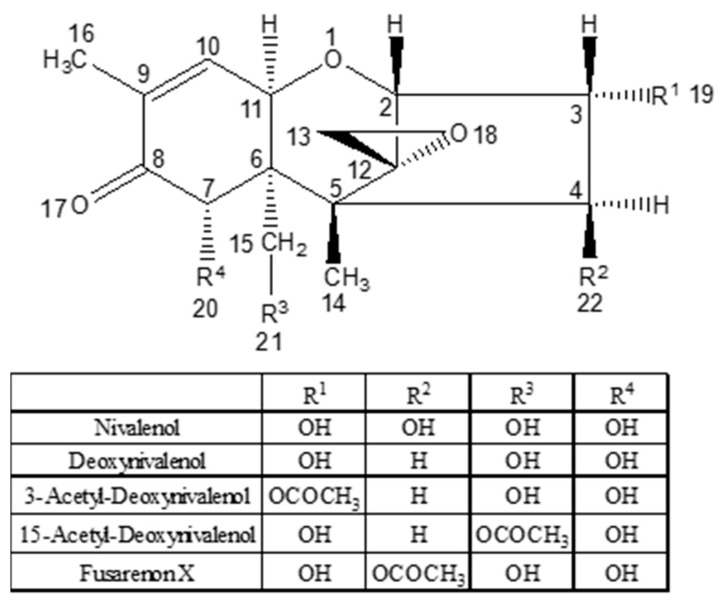
Generic structure of Type B-trichothecenes including deoxynivalenol (DON).

DON is one of a large family of trichothecene mycotoxins (Figure 7). The development of an exposure biomarker for DON by quantifying urinary free-DON and a DON-glucuronide combined as total urinary DON (T-DON) was suggested by observations in a rodent model [63] and there are pilot data from a handful of urine samples from China.

To better examine the utility of this putative urinary biomarker, three studies were conducted that examined the levels of urinary T-DON in UK adults in relation to diet [64,65,66]. Firstly, 25 paired urine samples were analyzed within a 7-day intervention study [64]. Individuals consumed their usual cereal-based diets for two days and on day 3, a first morning void was collected. On days 3–6, cereals were avoided; and on day 7, a further first morning void was collected. Food diaries revealed a highly significant *p* < 0.001 reduction in wheat and maize ingestion during the intervention phase, which was reflected in an 11-fold reduction in urinary T-DON; geometric mean level during normal diet (7.2 ng DON/mg creatinine; 95% CI 4.9–10.5); and post-intervention (0.6 ng/mg; 95% CI 0.4–0.9 ng/mg). A follow-up survey using the 2000–2001 UK adult National Diet and Nutrition Survey utilized 100 urine samples from each of a high-, moderate-, and low-cereal-consumption group. This analysis revealed a modest albeit significant relationship (*p* < 0.0005) between cereal intake and urinary T-DON [65]. An additional study of 35 individuals in which both urinary T-DON and cereal intake data were collected on each of six consecutive days confirmed these observation [66]. In that study, mean daily DON intake varied by approximately one log fold from just below 100 to approximately 1000 ng/kg bw/day. A more robust validation of the suggested urinary biomarker involved measuring T- DON intake for 25 UK adults on each of four consecutive days and the urinary T-DON concentration in the next morning. The mean DON intake over four days was significantly and strongly correlated with mean urinary T-DON (*p* < 0.001, R^2^ = 0.83) in multivariate analysis adjusting for age, sex and BMI. A strong correlation was also revealed when looking at individual day (*p* < 0.001, R^2^ = 0.56, 0.49, 0.54, 0.64, for each day respectively). Authors estimated that on average, 73% of the ingested DON was transferred to urine. Based on the strong quantitative relationship between exposure and the bio-measure, and the stability at both refrigerator and room temperature in the short term (24 h) and cryo-preservation in the long term (years), urinary T-DON was established as a useful exposure biomarker [66,67,68].

Studies by Warth and colleagues [69,70] and Vidal et al. [71] identified and confirmed that the major glucuronide in urine from individuals exposed to DON was 15-DON-glucuronide, and both free-DON and the glucuronide were transferred to urine. In a single-person study, approximately 68% of the DON was transferred to urine, measured as T-DON [70]; and in a multi-person study (*n* = 20), but at fixed dose using a bolus (1 ug/kg bw/day), DON was rapidly excreted within 24 h, and the recovered T-DON was 64.0 ± 22.8% of the dose [71]. Overall, approximately 65–75% of the ingested DON appears on average to be transferred to urine; this reported DON transfer contrasts sharply with the FB1 data. This difference may significantly contribute to a stronger dose–response relationship observed for the former even within a more limited range of exposure. It remains to be examined whether the ratio of DON:DON-glucuronide may represent a phenotypic measure of susceptibility to DON exposure (6, 67,68), or whether the original hypothesis from Turner and colleagues in reality simply reflects the difference in DON and DON-glucuronide toxicokinetics and timing of urine collection [71].

## 6. Ochratoxin A

**Figure 8:**
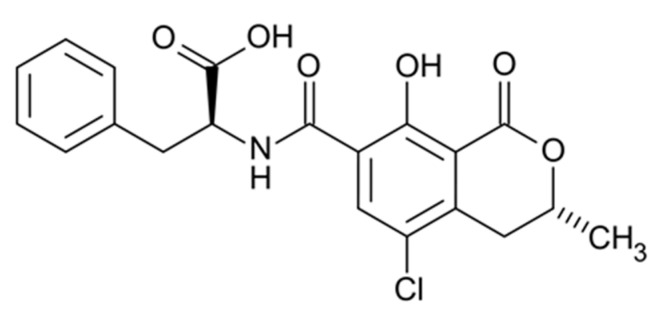
Structure of Ochratoxin A (OTA).

OTA (Figure 8) can contaminate a cornucopia of dietary staples through multiple fungal species in different climates throughout the globe [1,2,3,4,12,72], thus the availability of exposure biomarkers for epidemiological studies is particularly desirable. The toxicokinetics of OTA are complex; while rapidly absorbed, OTA is non-covalently associated with serum albumin, with a suggested <0.2% OTA free fraction in serum [73,74], slowing both its biotransformation and excretion half-life [75,76]. However, in humans, only sparse toxicokinetic data exist.

In a 2000 Swiss study, the serum and urine of one 57-year-old male volunteer was collected up to 75 days after consumption of a bolus of 3H-OTA [74]. Although the authors determined a half-life of 35.6 days for this single volunteer (a number frequently cited in literature), the data led the researchers to posit a compartmented model in which there is a shorter half-life of approximately 20 h within the first 6 days, and the longer half-life of 35.6 days thereafter.

Serum levels of OTA were measured in a group of 138 adults (age, 35–65 years) from the Tuscany region of Italy [77]. OTA was detected in 97% of the samples (mean and median, 560 and 480 pg/mL, respectively, with most data between 100 and 2800 pg/mL, and one extreme value at 57,200 pg/mL). A strong association was found with the season in which blood samples were obtained. A subgroup of subjects (*n* = 68) provided a repeat blood sample approximately one year later, and while overall mean levels were similar, no correlation was observed between paired samples. Based on the variation, OTA in serum was suggested as a potential marker of population-level rather than individual-level exposure. Although serum OTA has not yet been shown to have a good correlation with exposure, it remains frequently measured. The higher concentration of OTA in serum compared to urine means that it can be detected using less sensitive methods of analysis, and is a reliable qualitative indicator of exposure.

In a one-month-long study employing a duplicate diet method alongside analysis of weekly 24 h urine and serum samples, Gilbert et al. [78] quantified longer-term OTA exposure of 50 adults in the UK. Based upon OTA contamination in the food samples, researchers estimated an averaged OTA intake to a range of 260–3540 pg/kg bw/day. Composite serum and urine samples for each participant were created from the weekly samples for 50 samples total of each matrix. OTA contamination was identified in 100% of the serum samples (400 to 3110 pg/mL) and in 92% of the urine samples (<10 to 58 pg/mL). The study found a significant correlation (r = 0.52) between urinary OTA and dietary OTA intake (as determined by daily food diaries and samples), but not between serum OTA and dietary intake (r = 0.29); *p* values were not given. So, in contrast to serum, urinary OTA appears to correlate with estimated dietary consumption, but requires more complex and sensitive testing methods due to its much lower concentrations and the presence of OTA metabolites. Gilbert and colleagues expressed concern around the predictive value of these measures to provide strong quantitative estimates of intake.

## 7. Exposure Assessment in Young Children and Infants

The development and use of biomarkers to create improved exposure assessment have been largely confined to studies in adults, with only the intake of AF and previously suggested AF biomarkers examined in young children [30,32]. There is much interest in the susceptibility of the very young to mycotoxins, but there are limited data regarding the uptake and transfer of mycotoxins and their metabolites to breast milk, and the subsequent exposure and uptake in infants in this dietary form. The basic chemistry and toxicokinetics of the mycotoxins will impact both their uptake from the mother’s diet and their transfer to milk once ingested, thus one would predict AFs and OTA to be more frequently transferred to breast milk, and FB1 and DON poorly transferred. Aflatoxins are very lipid soluble and the AFB1 metabolite AFM1 dominates, though all AF parent compounds can potentially transfer to milk; the transfer is believed to represent less than approximately 3% of that AF ingested by mother (reviewed in depth by Degan et al. [79]). One study in Egypt estimated a milk to plasma (M/P) ratio for AFM1 of 0.21 [80], though blood AFM1 concentrations following maternal exposure are cleared rapidly limiting the strength of this ratio estimate. In one study of Gambian infants, AF-alb was only detected in the sera at 16 weeks of age, where the mothers had initiated complementary feeding prior to the blood draw, this occurred for 13 of 118 infants [81]. This observation is notable in that it indicates that breast feeding appears highly protective against aflatoxin exposure in a region where diet is frequently contaminated at high levels. These data reflect both the level of AF contamination of family/complementary food compared to that of breast milk where maternal diet is contaminated; but also the limit of detection of the assay. Thus, authors do not claim that there was no AF exposure at all in those fully breast feeding, but rather that it is not apparent via the established AF-alb biomarker. AFM1 being the major toxic AF in the diet of a breastfeeding infant, as opposed to the AFB1 in family foods, raises the query as to whether the AF-alb biomarker would capture this exposure. Structurally, AFM1 has the requisite double bond such that an 8,9-epoxide could be envisaged (compare structures in Figure 1, Figure 2 and Figure 3). However, neither the toxicokinetics of AFM1 nor a putative AFM–alb is established. The assay in these Gambian infants was an indirect ELISA (discussed elsewhere). The assay would not capture free AFs in serum, but if formed, it has possible but unproven potential for cross-reactivity with other aflatoxin-based amino acid adducts, which could include AFM–lysine from a suggested AFM–alb adduct. However, overall, breast feeding is thought to provide significant protection from aflatoxin exposure in comparison to complementary foods in mycotoxin-prone settings simply based on relative dose [6,10,79,82]. Neither the dose–response relationship between intake and the biomarker for this age group, nor the comparative nature in terms of risk for an AF-alb measure at 16 weeks versus older children has been examined to our knowledge. In the Gambia, AF-alb has additionally been reported in cord blood samples taken at birth, data strongly indicative of AFB1 transfer from maternal diet, and in utero exposure and biotransformation [81,83]. Mean cord blood levels of AF-alb were approximately 8-fold lower than the maternal blood [81], though this in no way should be translated to a 8-fold lower risk in the neonate.

The rapid bioavailability and high serum concentrations frequently seen in serum OTA suggest a potential for transfer to breast milk. Muñoz et al. [84] tracked the lactational transfer of OTA from the serum of 21 lactating Chilean mothers to their breast milk; parallel urine samples from their breastfeeding infants allowed the full exposure path from mother to infant to be monitored. Over the 6 month study period, serum, breast milk (including samples with colostrum), and infant urine samples were collected and analyzed. OTA was detected in 43 of the 45 plasma samples, ranging from the LOD (70 pg/mL) to 639 pg/mL. Of the mothers with OTA-positive serum results, 23 of the 37 breastmilk samples also tested positive for OTA, with calculated M/P ratios ranging from 0.01 to 0.86. Intra-individual variations were observed as well, with samples containing colostrum having a significantly higher average M/P ratio than that of mature milk (0.40 ± 0.26 vs. 0.26 ± 0.19 respectively, *p* = 0.001). The authors theorized that higher protein content of milk given during the first days’ post-partum, in which colostrum is present, may increase the OTA content during this crucial breastfeeding time. A strong correlation (r = 0.57) between breast milk OTA and infant urinary OTA consumption was also observed. Thus, as with the adult urinary OTA results, infant urinary OTA levels serve as a useful means of exposure estimation.

Biasucci et al. [85] used the EPIC questionnaire to determine OTA intake of 130 women in Italy (Italians and foreign nationals), and compared it to the cord serum and breast milk concentrations as determined by HPLC–FD. OTA was detected in 129 of the serum samples and 45 of the breast milk samples, with concentrations of positive samples ranging from 84 to 4835 pg/mL, and 1.1 to 75.1 pg/mL, respectively. When only positive samples were statistically analyzed, a positive serum/milk correlation was found (r = 0.53; *p* < 0.001). However, this did not remain when using all samples (r = 0.12, *p* > 0.05).

One study in Tanzania reported frequent FB1 in breast milk samples (58 of 131), with several extremely high values. Given that FBs are poorly absorbed, this is surprising and may reflect standards used in the quantification being unstable in methanol. This observation, however, merits further investigation, as it potentially predicts extremely high maternal and infant exposure on some occasions [86].

Several questions remain unanswered. Firstly, higher-quality data are needed on the relationship between maternal exposure and breast milk transfer for a range of toxins (discussed by Warth et al. [82]). Secondly, the relationship between maternal breast milk levels and infant urine warrants further investigation. It is more likely that infant urine will be collected in epidemiological studies, and both the infant uptake and biotransformation capacity are poorly defined. Too frequently assumptions used are based on adults. Thirdly, how are these processes modified during the transition through complimentary foods, a time when significant intestinal maturation occurs and when biotransformation capacity is rapidly changing? Finally, for aflatoxin exposure, it may be valuable to explore AFM–alb formation within early life settings as a potential additional contribution.

## 8. Biomarker Interpretation

In recent decades, there has been significant momentum to better understand exposure assessment for use in epidemiology, biomonitoring, and assessing the efficacy of interventions to mitigate exposures. Dose–response relationships have been established for only a few mycotoxins, with the most well established used for aflatoxins. The following provide assessment of exposure: for AFs, urinary AF-N7-Gua, AFM1 and serum AF-alb; for FBs, urinary FB1; for DON, urinary T-DON; for OTA, urinary OTA. For FBs, a combination of urinary FB1 and red blood cell Sa-Pi/So-Pi is strongly informative, while serum OTA provides a qualitative measure of exposure. There are multiple analytical approaches to measuring these biomarkers and the majority include either a form of immunoassay or separation by HPLC and detection by either fluorescence or an increasing array of mass spectrometry tools. The gold standard in terms of analytical specificity and sensitivity is typically LC with tandem mass spectrometry (LC–MS/MS). However, the high cost of purchasing and maintenance of equipment severely restrict such approaches to wealthier research laboratories and impair the capacity for many research groups from developing countries to truly control analytical research within their own country. However, there are strong HPLC methods and multiple well-developed commercially available immunoassays for several of the discussed biomarkers. A more important restriction in biomarker measures is the commercial availability of reagents with which to conduct the research. AFM1, DON, FB1 and OTA are readily available and the increasing availability of radiolabeled versions has been particularly valuable as mass spectrometry approaches now dominate the literature. However, for aflatoxins, the availability of relevant standards, with the exception of AFM1, is a major restriction, and AFM1 captures neither the measure of DNA damage obtained from using AF-N7-Gua nor provides the long-term measure obtained by AF-alb analysis. Instead, several research groups have independently undertaken a significant burden in chemical synthesis and purification of these analytes. Both require the activation of aflatoxin [87] followed by controlled adduction to generate either AF-N7-Gua for the urinary assay, or both mono AF-alb and the production of the AF-lys [14,15,20,88,89,90]. The lack of availability means that many interested research groups do not use either of these established biomarkers. Commercially available AFB1–albumin is not a suitable standard for digestion within the AF-alb assay as it is multiply adducted, estimates at 8–12:1 molar ratio of AFB to albumin on unspecified amino acids, and is simply not established as reflective of the adduct distribution in vivo, as 1:1 on lysine specifically within the albumin.

Another limitation in all the biomarker research conducted to date is the lack of comparative data between groups and methods. Two laboratories reported comparative data for both urinary FB1 and T-DON using samples from 55 females obtained from South Africa [91]. Approach-A used a multi-mycotoxin assay involving sample isolation and enrichment ahead of LC–MS [92], while Approach-B used two individual measures, one for T-DON and separately for FB1 [56,65]; both involved enrichment and LC–MS to quantify. For FB1, the mean concentration was somewhat higher for Approach-A (0.8 ng/mL, SD 1.1; range 0.02–4.9 ng/mL) compared to Approach-B (0.2 ng/mL, SD 0.2; range 0.01–1.3 ng/mL), and overall data were poorly correlated (r = 0.17, *p* = 0.25). For T-DON, although the mean data from the single-toxin approach (Approach B) was slightly higher than Approach-A, a highly significant correlation between the two approaches was observed (r = 0.94, *p* < 0.0001). These data suggest that DON data may be more comparable, while FB1, in comparison, is more limited, when evaluating studies and when using these specific assays. If alternative assays are used by other groups, these additionally need to be compared. The above authors additionally revealed using a third method [93], with lower sensitivity, that the dominant DON metabolite in the urine of these 55 south African women was a DON-15-glucuronide, rather than a DON-3-glucuronide [91]. A further comparison of urinary T-DON involved paired measures from 256 Swedish adults using the same analytical method for the singleton assay [65], but a slightly modified assay for the multi-mycotoxin approach described above, from the same laboratory [94]. In this survey, data were strongly correlated (r = 0.59, *p* = 0.0001), though overall the relationship for urinary T-DON was not as strong as the smaller study.

Three comparative studies were undertaken for the AF biomarker AF-alb. In the first, undigested AF-alb was quantified (direct ELISA) and data compared with analysis of the protease digest products from AF-alb using both an established in-house immunoassay (indirect ELISA) [95] and additionally an HPLC-fluorescence (HPLC–FL) assay [25]. Initially, data from rats dosed with radiolabeled AFB1 across a 400-fold range were examined. All three assays gave dose–response effects when compared to the radiolabeled measure of AF-alb, though the digest ELISA was most sensitive in terms of absolute detection, at 5 pg AFB-lys/mg of albumin. The direct ELISA was deemed as useful only as a screen for very high exposures, while the indirect ELISA and HPLC–FL were able to measure both moderate and high exposures. When 15 samples from persons from Kenya were analyzed using the two proteinase-based methods, there was a strong correlation between the data (r = 0.97) for samples with an approximate 2 log range of adduct. It was noted that data for AF-alb from the HPLC–FL were approximately 10-fold lower on average compared to the indirect ELISA. This observation may reflect differences in analyte recovery, but additionally the ELISA is likely to capture both incomplete AF-digest products from AF-bound albumin, perhaps as di- or tri-peptides including lysine-bound AF, whereas HPLC–FL would specifically only identify the AFB1-lysine produced. Authors also hypothesized that AFG1 and AFM1 adducts could be formed, and if so, they may only be captured by the ELISA (see also LC–MS discussion on this below).

The second study [96] conducted a quantitative comparison of AF-alb using a recently developed isotope dilution mass spectrometry (IDMS) [97] and the previously discussed indirect ELISA [95]. These assays compared data from 25 Guinean blood samples with a just over a one log variation in exposure estimates. The data from these assays were highly correlated (r = 0.88 m *p* = 0.0001). It was noted within this moderate exposure range (3–60 pg/mg (based on the ELISA) that on average, the ELISA data gave a 2.6-fold greater adduct concentration, again possibly related to the ELISA being able to measure incomplete or additional digest products of aflatoxin exposure. A more comprehensive comparison was made across a wider range of exposure using the indirect ELISA, the IDMS and an HPLC–FL assay [98], with improved sensitivity compared to [25]. Serum samples from an aflatoxicosis outbreak in Kenya (*n* = 102) with exposure spanning a multiple-log range were used, and LODs for the assays were ELISA of 3 pg/mg albumin, IDMS of 0.25 pg/mg, and by HPLC–FL 9 pg/mg. In this study, the sera measures ranged from 18 to 67,000 pg/mg, 2 to 17,700 ng/mg and non-detectable to 13,600 pg/mg, respectively. Linear regression slopes for the HPLC–FL versus IDMS, ELISA vs. IDMS and HLPC–FL versus ELISA were 0.70 (r^2^ = 0.95), 3.2 (r^2^ = 0.96), and 4.31 (r^2^ = 0.90), respectively. In a sub-analysis using only data below 500 pg/mg (*n* = 39), the regression coefficient for ELISA versus IDMS was 2.5 (r^2^ = 0.86). Overall, these three assays appear in good agreement and all are well suited for epidemiological study. Again, both of the physico-chemical methods gave on average slightly lower AF-alb estimates compared to the ELISA, most likely linked to additional adducts being formed but only being recognized by the indirect ELISA. An AFG1–albumin adduct has been identified [99], but remains poorly explored. It will be interesting to see whether recent significant advances in LC–MS approaches [97,100,101,102,103] to improve accuracy and capacity for mycotoxin bio-measures will further expand to better estimate the potential contribution of additional adducts. The potential use of dried blood samples, recently used for other mycotoxin biomarkers [104], could support a greater throughput and array of multi-AF-albumin measures, in addition to their value in OTA analysis in blood samples.

For both of the latter AF-alb studies and the DON and FB comparisons mentioned above, the compared methods were conducted in distinct laboratories and separate countries for some analyses, so the correlations (or lack thereof) between laboratories and methods can be informative concerning the reliability of comparative interpretations of studies using the specified analytical methods and biomarkers. Thus, the use of comparative data is valuable as researchers and policy makers try to assess and compare distinct studies. However, there is the danger of over interpretation within such comparisons that may in part be driven by the high level of accuracy apparent within some of the quantitative tools. These analytical techniques are invaluable in that they obtain precise and accurate data, and significantly reduce false contributions to the exposure estimate, but they need to be interpreted with respect to the noise or variation in the dose–response evaluations used from these biomarkers to make intake estimates. For example, the strength of the relationship between aflatoxin or fumonisin intake and any given biomarker measurement was typically obtained using studies across a three log range of exposure. Some of the better studies included both 95% CIs and occasionally margins of interpretation of these regression lines. Thus, the sensitivity, specificity and multi-decimal point accuracy, sometimes reported, for the concentration of the biological measure could or perhaps should be interpreted as representing a somewhat wider range in probable toxin intake rather than the point estimate that could be inferred from the laboratory analysis. This has several implications. Firstly, in designing epidemiological studies, researchers may need to better control for this implicit noise in what biomarker data represents; perhaps incorporating more stringent power calculations, and possibly simply requiring larger study numbers. This may be especially important for FB measures as proposed by Riley and colleagues [59,60,62]. Secondly, and again in relation to AF and FB measures, where the exposure data include only a very small range, perhaps those with significantly less than a log variation, then it may become problematic to infer biological effects associated with these changes even if data can be statistically modelled. This component includes both limits within sample timing, collection and extraction, as described by Gange et al. [105], and the fact that if a given study, for example, perhaps has data in the range 2–6 pg/mg, it does not necessarily mean very much in terms of how one should interpret the exposure in the lower quartile of that dataset versus the upper quartile.

The comparative studies for AF-alb [96,98] are particularly interesting in how data may end up being interpreted. The latter two comparisons discussed indicate that there are multiple tools to measure AF exposure that are useful. It may be tempting to take data from a novel epidemiological survey where exposure used an LC–MS/MS assay and perhaps make an adjustment to account for this suggested difference for an epidemiological study using the indirect ELISA, either by approximately 2.5- or 2.6-fold multiplication [96,98]. For the groups that established the relationships, this seems reasonable. For groups outside of this network, caution may be required. None of the standards used in these assays are commercially available, thus in-house production of the standards is required, with typically more than one method of synthesis. Such standards may be produced in mmol or nmol quantities, but then diluted to pmol and fmol for in assay use, so unless there is active collaboration between groups, these standards may not perfectly align. Perhaps when researchers wish to compare datasets, it may be more informative to think of how the data are more broadly stratified, with perhaps consideration of data being low, moderate, high and very high. The health effects associated with exposure are also likely to be setting related and thus other factors may significantly modify the biomarker level, and could modify the threshold exposures of observed adverse effects.

Finally, there is an increasing trend to further lower LOD of assay capability, thus we are increasingly likely to measure exposures that may have a large range of exposure but remain negligible or non-informative in biological importance. Thus, simply identifying a wide range of biomarker concentrations would not necessarily be sufficient to conduct a meaningful epidemiological investigation. To date, no studies have investigated the relationship between very low AF intake in humans and AF-alb, nor settings where exposure is intermittent, and thus very low AF-alb biomarker data alone need cautious interpretation in epidemiological studies.

## 9. Mycotoxin Biomarkers Summary

**Figure 9:**
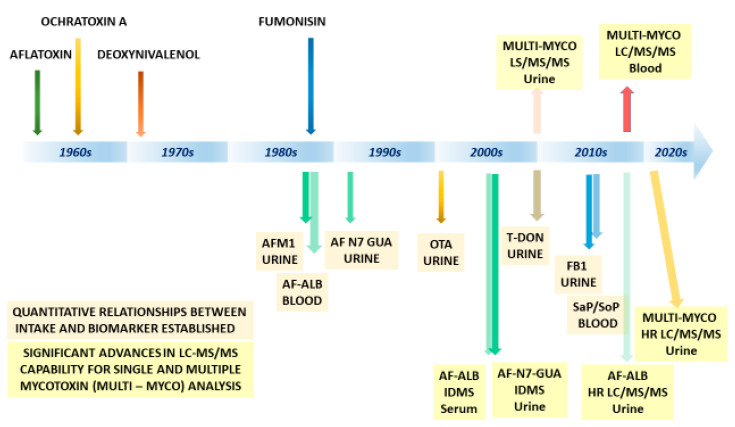
Timeline for identification and biomarker validation. AFM1—aflatoxin M1, AF-N7-GUA—aflatoxin N7-guanine, AF-ALB—aflatoxin–albumin, OTA—Ochratoxin A, T-DON—total deoxynivalenol, FB1—fumonisin B1, SaP/SoP—spinganine 1-phosphate/sphingosine 1-phosphate, IDMS—isotope dilution mass spectrometry, and HR—high resolution.

Mycotoxins are frequent contaminants of cereal crops throughout the world. The heterogeneous contamination and the homogeneous diets in regions at greatest risk of exposure lead to chronic exposure at high levels in many developing countries. Moderate chronic exposure to Fusarium mycotoxins in more temperate regions is also apparent. Mycotoxins can cause acute toxicity affecting 100 s to 100,000 s of individuals on occasions; and are fatal at high doses. Some mycotoxins are classified as carcinogenic or possibly carcinogenic, thus chronic exposure to aflatoxin, for example, is a major contributor to the burden of liver cancer [4,7]. The use of aflatoxin biomarkers described here was critical to confirming this family of toxins as proven human carcinogens [4,7,8,10]. An even greater burden of morbidity, and perhaps mortality, is now predicted from studies using aflatoxin biomarkers to assess immune effects and growth [4,10,11]; though these subtler effects are perhaps more complex to quantify. Other mycotoxins including those discussed here are likely to contribute to the overall disease burden, and biomarkers of exposure and effect will contribute to understanding the molecular epidemiology of mycotoxins in chronic disease. They will also support our understanding of approaches to intervene to restrict exposure. Figure 9 provides a rough timeline from mycotoxin identification to biomarker validation; in addition, several significant developments in some of the analytical tools have been highlighted with respect to LC–MS/MS. Table 1 summarizes the individual bio-measures and biomarkers discussed. These exposures also do not occur in isolation, and study design needs to better accommodate this, rather than simply collecting an ever-increasing diversity of measures.

## 10. Key Points

- Mycotoxins are an unavoidable component in many diets, especially in developing-world regions.
- Animal data concerning the toxicity of mycotoxins are clear and consistent, while our understanding of mycotoxin-induced human disease is limited.
- The development and use of validated exposure biomarkers confirmed the role of aflatoxins in the etiology of primary liver cancer and cirrhosis and will support the understanding and mitigation efforts linked to growth faltering.
- Urinary fumonisins and phosphorylated sphingoid bases in blood are good measures of FB1 exposure, though care is needed in epidemiological study design to more clearly demonstrate health effects.
- Urinary T-DON is strongly correlated with DON intake, and this exposure biomarker awaits application in epidemiological studies. Growth faltering and immune effects will likely dominate these studies.
- The further development and use of OTA exposure measures, perhaps with better recognition of their metabolite profiles, is crucial to understanding their role in human disease.
- The use of powerful LC–MS/MS approaches to understand exposure to multiple mycotoxin species has accelerated in the past decade or so, recently reviewed in detail [106]. For most, analytical quantification of their bio-fluid concentration has far outpaced good quantitative data on how to interpret these data. This is an important next step if we are to maximize these tools to perform public health good.
- The establishment of a biomarker database that captures in a uniform way or multiple ways would be invaluable.
- The continued expansion of collaborative studies that compare analytical tools and share analytical standards will greatly benefit a more comprehensive approach for biomarker-driven epidemiology of the mycotoxins of mycotoxin exposure and their health consequences.

## Figures and Tables

**Table 1:** Exposure bio-measures and biomarkers summary.

	Matrix	Dose Transferred	Relevant Time Frame	Validated	Standards Commercial
**Aflatoxin**					
AFM1	Urine	1–3%	24–48 h	Yes	Yes
AF-N7-Gua	Urine	1%	24–72 h	Yes	No
AFB-alb	Serum/plasma	1–3%	2–3 months	Yes	No
AFG-alb	Serum/plasma	n/e	n/e	No	No
**Deoxynivalenol**					
T-DON	Urine	65–75%	24–48 h	Yes	Partial *
DOM-1	Urine	<5%	24–48 h ^#^	No	Yes
**Fumonisin**					
Sa/So	Urine	Not relevant	n/e	No	Yes
Sa/So	Serum	Not relevant	n/e	No	Yes
FB1	Urine	<1%	24–72 h ^#^	Yes	Yes
Sa-Pi/So-Pi	Blood	Not relevant	n/e	Yes	Yes
**Ochratoxin A**					
OTA	Serum	n/e	Several weeks	No	Yes
OTA	Urine	n/e	?	Partial	Yes

AFM1—aflatoxin M1, AF-N7-Gua—aflatoxin N7-guanine, AF-alb—aflatoxin–albumin, T-DON—DON plus DON-glucuronide, DOM-1—de-epoxydeoxynivalenol, Sa/So—sphinganine/sphingosine ratio, FB1—fumonisin B1, Sa-Pi/So-Pi—spinganine-phosphate/sphingosine-phosphate ratio, #—predicted but not demonstrated, and n/e—not established. * DON is available but not DON glucuronides.

## Data Availability

No new data were created or analyzed in this study. Data sharing is not applicable to this article.
